# Plasma neurofilament heavy chain is a prognostic biomarker for the development of severe epilepsy after experimental traumatic brain injury

**DOI:** 10.1111/epi.18149

**Published:** 2024-10-14

**Authors:** Mette Heiskanen, Ivette Banuelos, Eppu Manninen, Pedro Andrade, Elina Hämäläinen, Noora Puhakka, Asla Pitkänen

**Affiliations:** ^1^ A. I. Virtanen Institute for Molecular Sciences University of Eastern Finland Kuopio Finland

**Keywords:** enzyme‐linked immunosorbent assay (ELISA), fluid percussion injury, posttraumatic epilepsy, rat, ROC curve analysis

## Abstract

**Objective:**

This study was undertaken to test whether the postinjury plasma concentration of phosphorylated neurofilament heavy chain (pNF‐H), a marker of axonal injury, is a prognostic biomarker for the development of posttraumatic epilepsy.

**Methods:**

Tail vein plasma was sampled 48 h after traumatic brain injury (TBI) from 143 rats (10 naïve, 21 controls, 112 with lateral fluid percussion injury) to quantify pNF‐H by enzyme‐linked immunosorbent assay. During the 6th postinjury month, rats underwent 30 days of continuous video‐electroencephalographic monitoring to detect unprovoked seizures and evaluate epilepsy severity. Somatomotor (composite neuroscore) and spatial memory (Morris water maze) testing and quantitative T_2_ magnetic resonance imaging were performed to assess comorbidities and lesion severity.

**Results:**

Of the 112 TBI rats, 25% (28/112) developed epilepsy (TBI+) and 75% (84/112) did not (TBI−). Plasma pNF‐H concentrations were higher in TBI+ rats than in TBI− rats (*p* < .05). Receiver operating characteristic curve analysis indicated that plasma pNF‐H concentration distinguished TBI+ rats from TBI− rats (area under the curve [AUC] = .647, *p* < .05). Differentiation was stronger when comparing TBI+ rats exhibiting severe epilepsy (≥3 seizures/month) with all other TBI rats (AUC = .732, *p* < .01). Plasma pNF‐H concentration on day 2 (D2) distinguished TBI+ rats with seizure clusters from other TBI rats (AUC = .732, *p* < .05). Higher plasma pNF‐H concentration on D2 after TBI correlated with lower neuroscores on D2 (*p* < .001), D6 (*p* < .001), and D14 (*p* < .01). Higher pNF‐H concentration on D2 correlated with greater T_2_ signal abnormality volume on D2 (*p* < .001) and D7 (*p* < .01) and larger cortical lesion area on D182 (*p* < .01). Plasma pNF‐H concentration on D2 did not correlate with Morris water maze performance on D37–D39.

**Significance:**

Plasma pNF‐H is a promising clinically translatable prognostic biomarker for the development of posttraumatic epilepsy with frequent seizures or seizure clusters.

**Key points**

- A first demonstration is made of plasma pNF‐H concentration as a biomarker for the development of severe posttraumatic epilepsy.
- Plasma pNF‐H concentration correlated with cortical lesion severity.
- Plasma pNF‐H concentration was associated with the evolution of epilepsy‐related comorbidities, correlating with somatomotor recovery but not spatial memory.

## 1 INTRODUCTION

Annually, approximately 69 million individuals worldwide experience a traumatic brain injury (TBI), mostly (~90%) mild TBI.^1^, ^2^ In addition, ~5 million people are diagnosed with epilepsy annually (World Health Organization, https://www.who.int/publications/i/item/who‐information‐kit‐on‐epilepsy). In approximately 40% of cases, epileptogenesis is initiated by structural causes such as TBI.^3^ Posttraumatic epilepsy (PTE) accounts for approximately 5% of all epilepsies and 10%–20% of structural epilepsies.^4^ Epidemiologic studies have demonstrated that the risk of epileptogenesis increases by up to 50% depending on the TBI severity and type, reaching 1%–4% even after mild TBI.^5^, ^6^, ^7^, ^8^, ^9^ Therefore, the overall number of subjects with an increased risk of epileptogenesis after TBI can be estimated as >1 million per year.

Approximately 20 hypothesis‐driven monotherapy approaches have demonstrated some disease‐modifying effects in models of posttraumatic epileptogenesis (i.e., development of epilepsy and progression after the condition is established).^10^ No clinical antiepileptogenic interventions are available for at‐risk persons, however, and their development remains a research priority.^11^, ^12^ One major obstacle to therapy development is the lack of prognostic/predictive biomarkers for epileptogenesis that could be used to stratify patient populations for antiepileptogenesis trials and serve as surrogate endpoints to reduce study costs, making sufficiently powered clinical trials affordable.^13^

Blood‐derived biomarkers in TBI have been extensively studied in animal models and humans.^14^, ^15^, ^16^, ^17^, ^18^ Their attractiveness relates to reduced invasiveness, easy and standardizable sampling that can be repeated several times over the course of disease development, availability of assay methodologies, and reasonable cost.^19^ Plasma/serum proteins and microRNAs have shown promise as diagnostic and prognostic biomarkers of TBI.^14^, ^15^, ^16^, ^17^ Studies to identify biomarkers for specific post‐TBI comorbidities, such as PTE, however, remain scarce.^10^, ^20^ These include plasma protein biomarkers such as neurofilament light chain (NF‐L); electroencephalographic (EEG) biomarkers, such as N3–rapid eye movement transition sleep spindles; high‐frequency oscillations and epileptiform discharges; and magnetic resonance imaging (MRI) biomarkers, such as severity of cortical damage.^10^ No candidate biomarkers identified in proof‐of‐concept studies have been verified in independent studies, however, and validated biomarkers for preclinical and clinical studies are lacking.

Neurofilaments are grouped into five filament families based on molecular mass.^21^, ^22^, ^23^ Neurofilament heavy chain (NF‐H) has the highest molecular weight, followed by medium chain, NF‐L, α‐internexin, and peripherin.^24^ Animal and human studies revealed that phosphorylated NF‐H (pNF‐H) is secreted in cerebrospinal fluid and blood after TBI.^25^, ^26^, ^27^ Histologic and MRI studies have shown that an increased concentration of neurofilaments, including pNF‐H, is associated with (myelinated) long‐distance axonal damage.^22^, ^28^, ^29^, ^30^, ^31^ Because pNF‐H is rather resistant to protease degradation, has prolonged postinjury expression in plasma, and can be assayed reproducibly, the plasma pNF‐H concentration has been extensively studied in experimental and clinical TBI and other brain diseases.^23^, ^32^ Based on these studies, plasma/serum pNF‐H exhibits promise as a diagnostic and prognostic biomarker for TBI in animal models and in humans, and as a predictive biomarker for therapy response.^33^, ^34^, ^35^ Recent cross‐sectional studies reported regulated nonphosphorylated NF‐H or pNF‐H concentration*s* in the brain and serum of patients with non‐TBI‐related structural epilepsies^36^, ^37^, ^38^, ^39^, ^40^ and in animals and humans with acute seizures.^41^, ^42^, ^43^, ^44^, ^45^, ^46^ Whether early changes in the pNF‐H concentration after TBI can be used as a biomarker for the risk of posttraumatic epileptogenesis, however, is unclear.

The present study was designed to test the hypothesis that acute post‐TBI plasma pNF‐H concentration is a prognostic biomarker for the development of experimental PTE. Data were derived from a large European Union FP7‐funded EPITARGET cohort of 10 naïve rats, 21 sham‐operated experimental controls, and 112 rats with severe lateral fluid percussion injury (FPI)‐induced TBI (www.epitarget.eu).^47^ Epilepsy phenotyping was performed during the 6th postinjury month with 1‐month video‐EEG (vEEG) monitoring. Plasma samples were collected 48 h after TBI. Behavioral and imaging data from the rats were used to analyze postinjury plasma pNF‐H concentration as a prognostic biomarker for PTE‐related comorbidities and cortical damage.

## 2 MATERIALS AND METHODS

**FIGURE 1:**
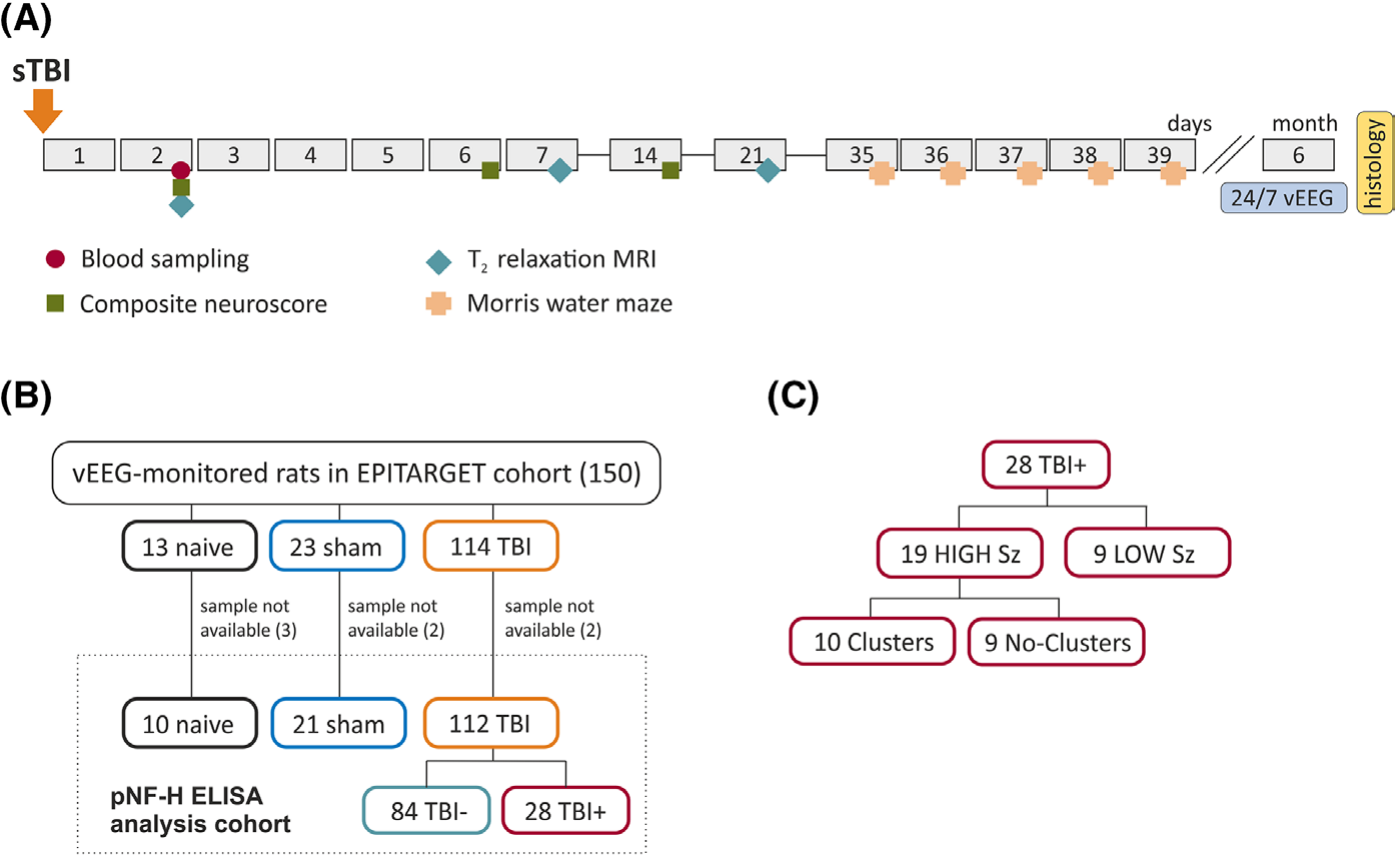
Study design. (A) Severe traumatic brain injury (sTBI) was induced with lateral fluid percussion‐induced TBI on day 0 (D0). Tail vein blood was collected for the phosphorylated neurofilament heavy chain (pNF‐H) analysis 48 h after TBI or sham operation. Naïve animals were sampled as controls. A composite neuroscore test was performed on D2, D6, and D14 to monitor somatomotor recovery. The Morris water maze test was performed on D35–D39 to assess spatial learning and memory. Quantitative T2 magnetic resonance imaging (MRI) was performed on D2, D7, and D21 to monitor the evolution of cortical pathology. Continuous video‐electroencephalographic (vEEG) recording was performed during the 6th postinjury month to monitor the occurrence of spontaneous late seizures (i.e., to diagnose posttraumatic epilepsy) and epilepsy severity. Finally, rats were perfused for histology to assess the cortical lesion area using unfolded cortical maps. (B) From the total of 150 animals that completed the vEEG monitoring,^47^ plasma samples were available from 143 rats (10 naïve, 21 sham, and 112 TBI [28 with epilepsy, TBI+; 84 without epilepsy, TBI−]) for analysis by enzyme‐linked immunosorbent assay (ELISA). (C) Epilepsy phenotype. Of the 28 TBI+ rats, 19 had high seizure frequency (HIGH Sz, ≥3 seizures per month) and nine had low seizure frequency (LOW Sz, <3 seizures per month). Of the 19 rats with high seizure frequency, seizures occurred in clusters (at least 3 seizures per 24 h) in 10 animals.

The study design and number of animals at different phases of the study are shown in Figure 1A. Materials and methods, ethics, and statistics are described in detail in supporting document Data S1. Detailed descriptions of the overall project and procedures were previously reported.^47^, ^48^

## 3 RESULTS

The final vEEG‐phenotyped EPITARGET cohort after histology‐based exclusions (abscesses) included 150 rats (13 naïve, 23 sham‐operated experimental controls, 114 rats with TBI). In the TBI group, the prevalence of epilepsy was 25% (29/114).^47^ The pNF‐H analysis cohort included 95% (143/150) of the total phenotyped EPITARGET cohort, that is, the rats from which the plasma samples were available for the enzyme‐linked immunosorbent assay (ELISA) analysis (10 naïve, 21 sham, 112 TBI; Figure 1B). Of the 112 TBI rats, 25% (28/112) had epilepsy (TBI+) and 75% (84/112) did not have epilepsy (TBI−).

Data on impact pressure, acute postimpact seizurelike behavior and apnea duration, and their association with plasma pNF‐H levels on day 2 (D2) and development of epilepsy are presented in supporting document Data S1.

### 3.1 Epilepsy phenotype

In the TBI+ group (*n* = 28), mean seizure number during the 30‐day monitoring period was 6.64 ± 5.82 (median = 4.50, range = 1–18). Of the 28 rats, 10 (36%) had seizure clusters (≥3 seizures/24 h) and 19 (68%) had ≥3 seizures during the 30‐day monitoring period (7 [25%] rats had one seizure and two [7%] had two seizures per month; Figure 1C).

### 3.2 Plasma pNF‐H concentration after mild (craniotomy) and severe TBI

#### 3.2.1 TBI and craniotomy increase plasma pNF‐H concentration

##### Naïve rats

**FIGURE 2:**
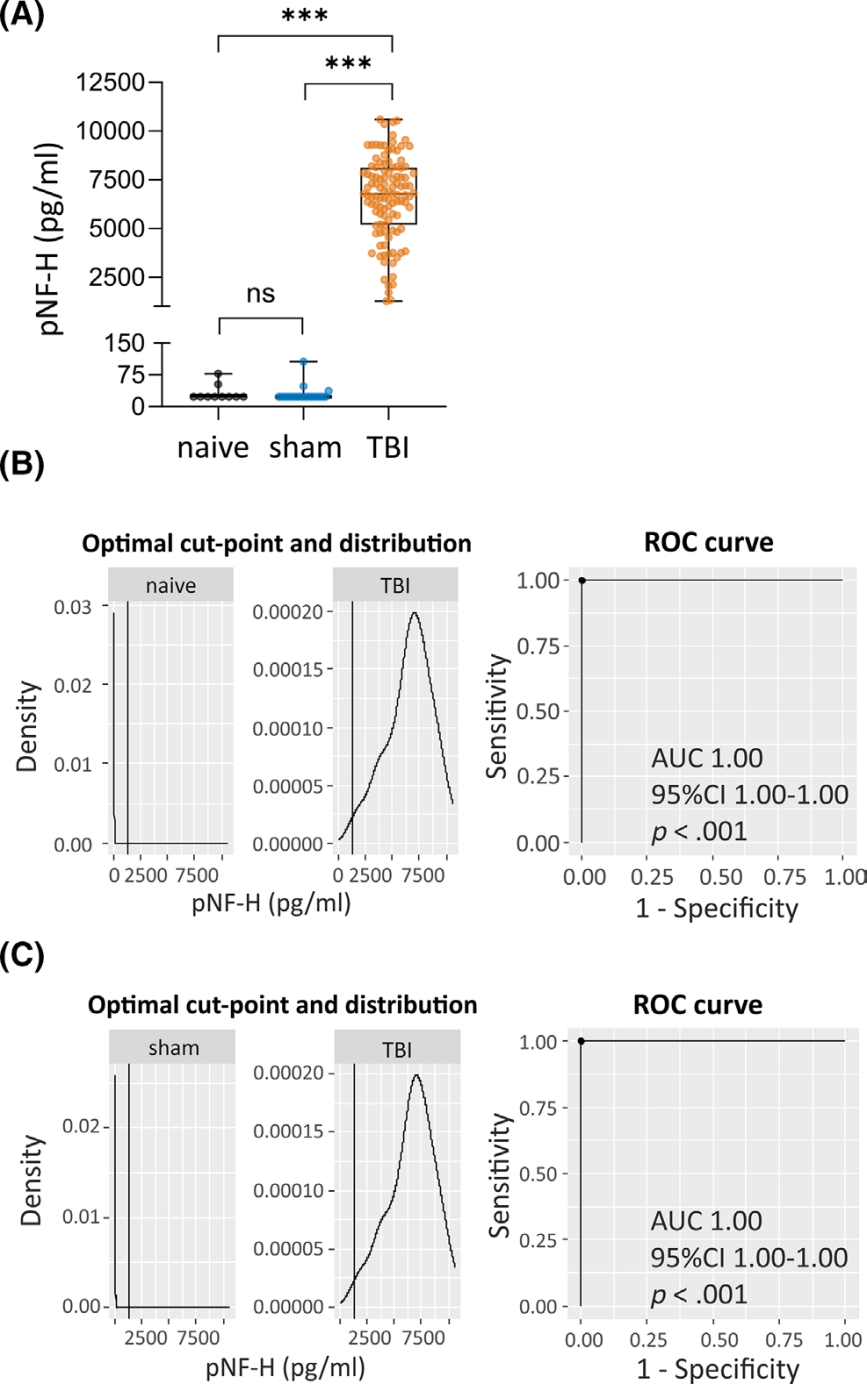
Plasma phosphorylated neurofilament heavy chain (pNF‐H) as diagnostic biomarker for traumatic brain injury (TBI). (A) Box and whisker plots (whiskers: minimum and maximum; box: interquartile range; line: median). Each dot represents one animal. pNF‐H concentration on day 2 (D2) was elevated in TBI animals (*n* = 112) compared with naïve rats (*n* = 10) or sham‐operated experimental controls (*n* = 21). (B) Plasma pNF‐H concentration on D2 distinguished TBI rats (*n* = 112) from naïve rats (*n* = 10) with 100% sensitivity and 100% specificity (area under the curve [AUC] = 1.00, 95% confidence interval [CI] = 1.00–1.00, *p* < .001; cutoff = 1269 pg/mL). (C) Plasma pNF‐H concentration on D2 distinguished TBI rats (*n* = 112) from sham‐operated experimental controls (*n* = 21) with 100% sensitivity and 100% specificity (AUC = 1.00, 95% CI = 1.00–1.00, *p* < .001; cutoff = 1269 pg/mL). ***p < .001 by Kruskal–Wallis test, followed by post hoc analysis using the Mann–Whitney *U* test. ns, not significant; ROC, receiver operating characteristic.

Only two of 10 plasma samples from naïve rats had pNF‐H concentrations above the limit of detection (LOD) of the ELISA (LOD = 23.5 pg/mL): 52.8 and 78.0 pg/mL (mean = 65.4 pg/mL). For subsequent statistical analyses, the pNF‐H concentration of the remaining eight samples with pNF‐H < LOD was set to 23.5 pg/mL. Thus, the average plasma pNF‐H concentration in samples from naïve animals was 31.9 ± 18.6 pg/mL (*n* = 10; Figure 2).

##### Sham‐operated experimental control rats

Only three of 21 samples collected on D2 (48 h after sham operation) had plasma pNF‐H concentration > LOD, varying from 37.3 to 106.5 pg/mL (mean = 64.1 pg/mL, median = 48.6 pg/mL). For the remaining 18 samples with pNF‐H concentration < LOD, the pNF‐H concentration was set to 23.5 pg/mL. Thus, the average plasma pNF‐H concentration in the sham‐operated control group was 29.3 ± 18.7 pg/mL (*n* = 21). The plasma pNF‐H concentration did not differ significantly between the naive and sham‐operated control samples (*p* > .05; Figure 2).

##### Traumatic brain injury

All 112 TBI samples collected on D2 had a plasma pNF‐H concentration > LOD (mean = 6588 ± 2134 pg/mL, range = 1269–10 595 pg/mL, median = 6768 pg/mL). TBI rats showed a 207‐fold increase in the plasma pNF‐H concentration compared with naïve rats (6588 ± 2134 pg/mL vs. 32 ± 19 pg/mL, *p* < .001) and a 225‐fold increase compared with sham‐operated controls (6588 ± 2134 pg/mL vs. 29 ± 19 pg/mL, *p* < .001; Figure 2).

#### 3.2.2 Plasma pNF‐H as diagnostic biomarker for TBI

We assessed whether plasma pNF‐H concentrations on D2 after TBI differentiated rats with TBI from naïve animals and/or sham‐operated controls.

##### TBI versus naïve

Receiver operating characteristic (ROC) curve analysis differentiated TBI rats from naïve rats with 100% sensitivity and 100% specificity (area under the curve [AUC] = 1.0, 95% confidence interval [CI] 1.0–1.0, *p* < .001; cutoff = 1269 pg/mL; Figure 2B).

##### TBI versus sham‐operated controls

ROC curve analysis differentiated TBI rats from sham‐operated control rats with 100% sensitivity and 100% specificity (AUC = 1.0, 95% CI = 1.0–1.0, *p* < .001; cutoff = 1269 pg/mL; Figure 2C).

### 3.3 Plasma pNF‐H as a prognostic biomarker for PTE

The prevalence of epilepsy in the pNF‐H analysis cohort was 25% (28/112), including 28 TBI+ and 84 TBI− rats. In the TBI+ group (*n* = 28), the mean seizure frequency was .210 ± .189 seizures/day (median = .129 seizures/day), mean seizure duration was 89 ± 34 s (median = 85 s), and mean Racine score was 2.8 ± 1.1 (median = 3.0).

#### 3.3.1 Posttraumatic epilepsy

**FIGURE 3:**
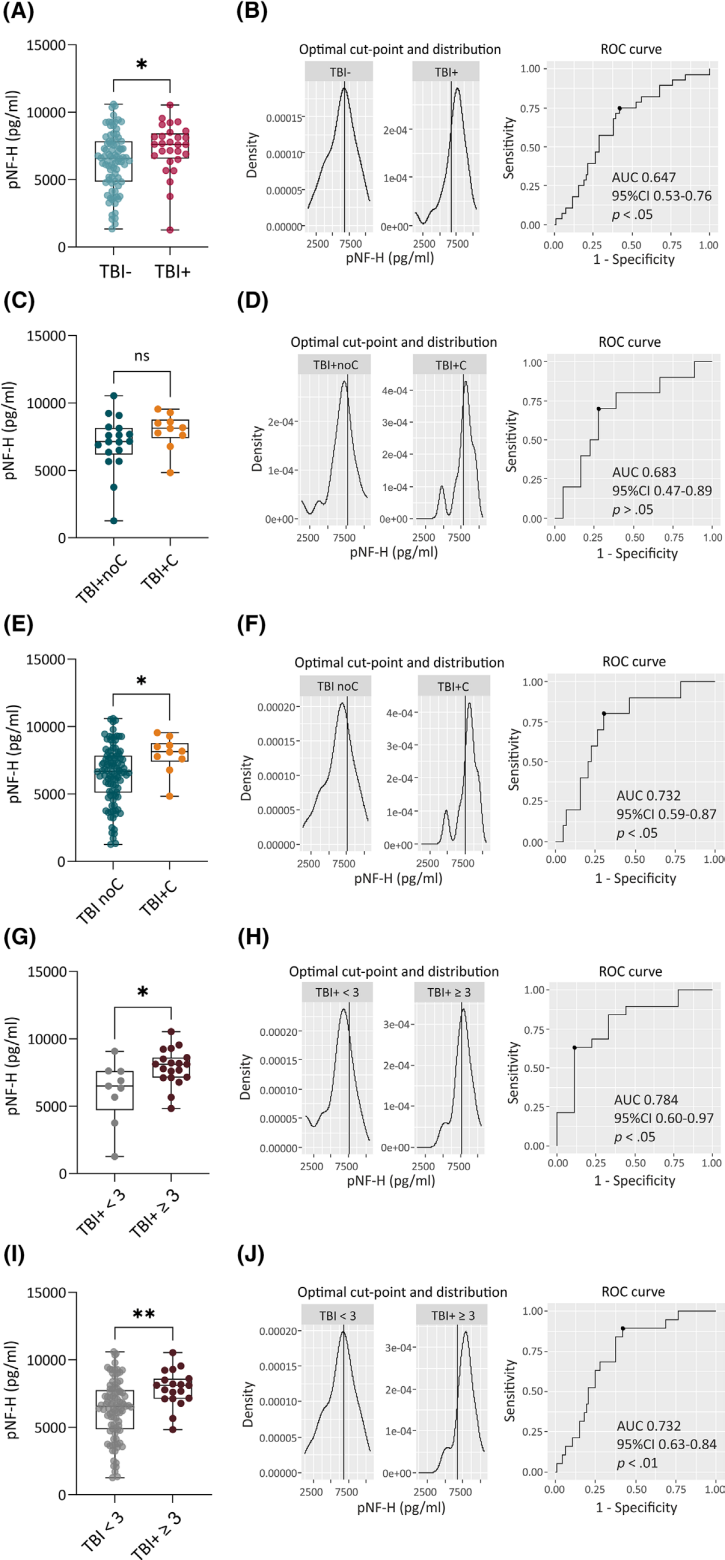
Plasma phosphorylated neurofilament heavy chain (pNF‐H), epileptogenesis, and epilepsy severity. (A) Plasma pNF‐H concentrations on day 2 (D2) were higher in rats with epilepsy (traumatic brain injury [TBI]+; *n* = 28) than in rats without epilepsy (TBI−; *n* = 84, *p* < .05). (B) Receiver operating characteristic (ROC) curve analysis indicated that plasma pNF‐H concentration differentiated TBI+ (*n* = 28) and TBI− (*n* = 84) animals (area under the curve [AUC] = .647, *p* < .05; cutoff = 6773 pg/mL). (C) In the TBI+ group, plasma pNF‐H concentrations were comparable between rats with seizure clusters (TBI+C; ≥ 3 seizures within 24 h, *n* = 10) or without seizure clusters (TBI+noC; *n* = 18, *p* > .05). (D) ROC analysis revealed that pNF‐H concentration did not differentiate between TBI+ rats with (TBI+C; *n* = 10) or without (TBI+noC; *n* = 18) seizure clusters (AUC = .685, *p* > .05). (E) In the whole TBI group, plasma pNF‐H concentrations were higher in TBI+ rats with seizure clusters (TBI+C; *n* = 10) than in all other TBI rats without clusters (TBI noC; *n* = 102; *p* < .05). (F) ROC analysis revealed that in the whole TBI group, plasma pNF‐H concentration differentiated TBI+ rats with clusters (TBI+C; *n* = 10) from all other TBI rats without clusters (TBI noC; *n* = 102; AUC = .732, *p* < .05; cutoff = 7600 pg/mL). (G) TBI+ rats that experienced ≥3 seizures during the 1‐month video‐electroencephalographic monitoring during the 6th postinjury month (TBI+ ≥3; *n* = 19) had slightly higher plasma pNF‐H concentrations on D2 than TBI+ rats with <3 seizures (TBI+ < 3; *n* = 9, *p* < .05). (H) ROC analysis revealed that plasma pNF‐H concentrations differentiated TBI+ rats with ≥3 seizures (*n* = 19) from TBI+ rats with <3 seizures (*n* = 9; AUC = .784, *p* < .05; cutoff = 7672 pg/mL). (I) In the whole TBI group, plasma pNF‐H concentrations in TBI+ rats with ≥3 seizures/month (*n* = 19) were higher than in all remaining TBI rats (TBI < 3; *n* = 93, *p* < .01). (J) ROC analysis revealed that in the whole TBI group, plasma pNF‐H concentrations differentiated TBI+ rats with ≥3 seizures/month (*n* = 19) from all other TBI rats (*n* = 102; AUC = .732, *p* < .05; cutoff = 6773 pg/mL). *p < .05, **p < .01 (Mann–Whitney *U*‐test). CI, confidence interval; ns, not significant.

Plasma pNF‐H concentration in the TBI+ group was 115% of that in the TBI− group (7311 ± 1881 pg/mL vs. 6347 ± 2168 pg/mL, *p* < .05; Figure 3A). ROC curve analysis indicated that the plasma pNF‐H concentration distinguished TBI+ rats from TBI− rats with 75% sensitivity and 58% specificity (AUC = .65, 95% CI = .53–.76, *p* < .05; cutoff = 6773 pg/mL; positive predictive value [PPV] = 38%, negative predictive value [NPV] = 88%, accuracy = 63%; Figure 3B).

#### 3.3.2 Severity of epilepsy

We assessed whether plasma pNF‐H concentrations differed depending on epilepsy severity (occurrence of seizure clusters, high seizure frequency).

##### Seizure clusters

Within the TBI+ group (*n* = 28), plasma pNF‐H concentration on D2 did not differ between TBI+ rats with (*n* = 10) or without (*n* = 18) seizure clusters (≥3 seizures within 24 h^49^; *p* > .05; Figure 3C, Figure S5A). ROC curve analysis indicated that plasma pNF‐H concentrations on D2 did not distinguish rats with (*n* = 10) and without (*n* = 18) seizure clusters (AUC = .683, 95% CI = .47–.89, *p* > .05; Figure 3D).

In the TBI group, however, TBI+ rats with seizure clusters had higher plasma pNF‐H concentrations than the remaining TBI rats (TBI+ animals without seizure clusters and TBI− animals, *n* = 102; 7920 ± 1350 pg/mL vs. 6458 ± 2156 pg/mL, *p* < .05; Figure 3E). ROC curve analysis indicated that plasma pNF‐H concentration distinguished TBI+ rats with seizure clusters (*n* = 10) from all other TBI rats (*n* = 102) with 80% sensitivity and 70% specificity (AUC = .732, 95% CI = .59–.87, *p* < .05; cutoff = 7600 pg/mL, PPV = 21%, NPV = 97%, accuracy = 71%; Figure 3F, Table S1).

##### Seizure frequency

Within the TBI+ group (*n* = 28), plasma pNF‐H concentrations were higher in TBI+ rats with ≥3 seizures/month (*n* = 19) than in TBI+ rats with <3 seizures/month (*n* = 9; 7895 ± 1336 pg/mL vs. 6079 ± 2325 pg/mL, *p* < .05; Figure 3G, Figure S5B). The higher the plasma pNF‐H concentration on D2, the greater the total number of late seizures during the 6th post‐TBI month (*ρ* = .467, *p* < .05; Figure S4). ROC curve analysis indicated that plasma pNF‐H concentration on D2 distinguished TBI+ rats with ≥3 seizures (*n* = 19) from TBI+ rats with <3 seizures (*n* = 9) with 63% sensitivity and 89% specificity (AUC = .784, 95% CI = .60–.97, *p* < .01; cutoff = 7672 pg/mL; PPV = 92%, NPV = 53%, accuracy = 71%; Figure 3H, Table S1).

In the TBI rats, plasma pNF‐H concentrations were higher in TBI+ rats with ≥3 seizures/month than in the remaining TBI rats (TBI+ animals with <3 seizures/month and TBI− animals, *n* = 93; 7895 ± 1336 pg/mL vs. 6321 ± 2172 pg/mL, *p* < .01; Figure 3I). ROC curve analysis revealed that plasma pNF‐H concentration on D2 distinguished TBI+ rats with ≥3 seizures (*n* = 19) from other TBI rats (*n* = 93) with 89% sensitivity and 58% specificity (AUC = .732, 95% CI = .63–.84, *p* < .01; cutoff = 6773 pg/mL; PPV = 30%, NPV = 96%, accuracy = 63%; Figure 3J, Table S1).

### 3.4 Plasma pNF‐H concentration and the severity of acute and chronic cortical damage

#### 3.4.1 Plasma pNF‐H and cortical T_2_ signal abnormality volume in MRI

##### Sham‐operated experimental controls

**FIGURE 4:**
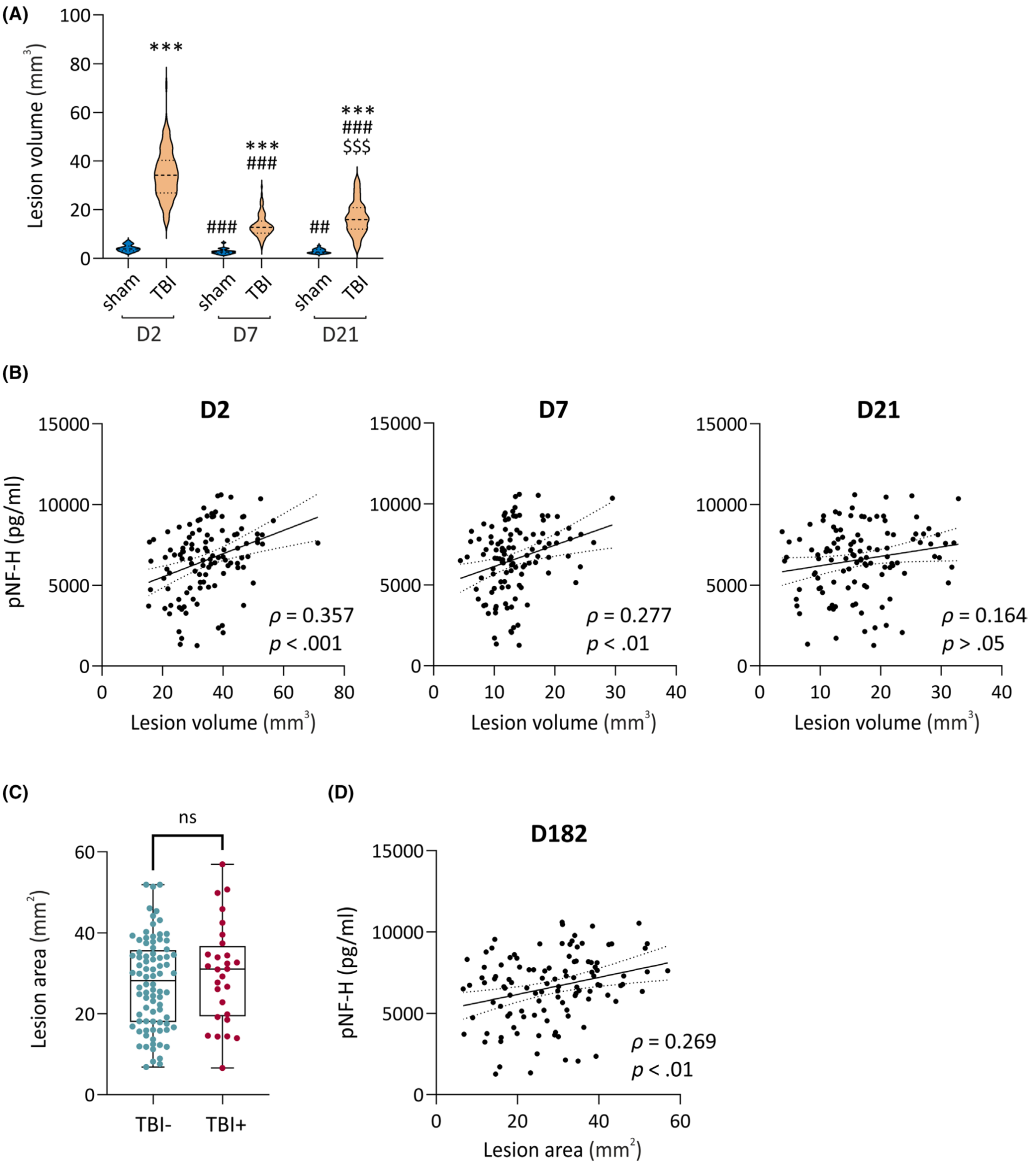
Plasma phosphorylated neurofilament heavy chain (pNF‐H) and cortical lesion severity on quantitative T2 magnetic resonance imaging (MRI; day 2 [D2], D7, D21) and in histologic analysis (D182). (A) Violin plots showing the total volume of abnormal voxels in rat brain MRI on D2, D7, and D21 after traumatic brain injury (TBI; *n* = 111 for D2, *n* = 112 for D7 and D21) or sham operation (*n* = 21). ***p < .001 for TBI compared to sham (Mann–Whitney *U*‐test); ##*p* < .01, ###*p* < .001 compared to D2; $$$*p* < .001 compared to D7 (Friedman test followed by post hoc Wilcoxon matched‐pairs signed rank test). (B) Spearman correlations (*ρ*) between the plasma pNF‐H concentration on D2 (y‐axis) and cortical lesion volume on MRI (TBI group only) on D2, D7, and D21 (x‐axis). Dotted lines indicate 95% confidence interval. (C) Box and whisker plots (whiskers: minimum and maximum; box: interquartile range; line: median) showing comparable cortical lesion area in TBI rats with (TBI+; *n* = 28) or without (TBI−; *n* = 84) epilepsy. (D) Spearman correlation (*ρ*) between plasma pNF‐H concentration on D2 and cortical lesion area on D182 after TBI (*n* = 112, *ρ* = .269). ns, not significant.

On D2, the cortical T_2_ signal abnormality volume was small (4.1 ± 1.4 mm^3^; Figure 4A). On D7, it was reduced to 2.8 ± 1.1 mm^3^ (D7 vs. D2, *p* < .001), and on D21, it was 3.0 ± .9 mm^3^ (D2 vs. D21, *p* < .01; D7 vs. D21, *p* > .05).

As only three sham‐operated rats had plasma pNF‐H concentrations > LOD of the ELISA, the correlation between pNF‐H concentration and cortical T_2_ signal abnormality volume could not be assessed. In the three sham‐operated rats with elevated plasma pNF‐H concentrations, the T_2_ signal abnormality volume was 3.8 mm^3^ (pNF‐H = 107 pg/mL), 7.0 mm^3^ (pNF‐H = 37 pg/mL), and 5.9 mm^3^ (pNF‐H = 49 pg/mL) on D2; 2.3, 4.2, and 2.7 mm^3^ on D7; and 3.2, 5.1, and 2.2 mm^3^ on D21. The sham‐operated rat with a pNF‐H concentration of 37 pg/mL on D2 had a T_2_ signal abnormality volume in the upper 25% percentile at all three time points; the rat with a pNF‐H concentration of 49 pg/mL had a T_2_ signal abnormality volume in the upper 25% percentile on D2, but not at other time points; and the sham‐operated rat with the highest pNF‐H concentration (107 pg/mL) had a signal abnormality volume between the lower and upper 25% percentiles at all time points.

##### Traumatic brain injury

On D2, the mean cortical T_2_ signal abnormality volume was 34.6 ± 10.0 mm^3^ (Figure 4A). By D7, the edema had resolved, and the T_2_ signal abnormality volume decreased to 13.4 ± 4.5 mm^3^ (D7 vs. D2, *p* < .001). By D21, the T_2_ signal abnormality volume slightly increased to 16.7 ± 6.8 mm^3^ (D21 vs. D7, *p* < .001).

The higher the pNF‐H concentration, the greater the T_2_ signal abnormality volume on D2 as well as on D7 (D2, *ρ* = .357, *p* < .001; D7, *ρ* = .277, *p* < .01; Figure 4B). No correlation was detected between the plasma pNF‐H concentration on D2 and the T_2_ signal abnormality volume on D21 (*ρ* = .164, *p* > .05).

#### 3.4.2 Plasma pNF‐H and cortical lesion area in histologic sections

Cortical lesion area in unfolded maps prepared from histologic sections on D182 after TBI ranged between 6.6 and 56.9 mm^2^ (mean = 28.2 ± 11.5 mm^2^, median = 29.3 mm^2^). Cortical lesion area did not differ between the TBI+ (*n* = 28, 30.0 ± 12.4 mm^2^) and TBI− (*n* = 84, 27.6 ± 11.2 mm^2^) rats (*p* > .05; Figure 4C). The higher the plasma pNF‐H concentration on D2, the larger the cortical lesion area on D182 (*ρ* = .269, *p* < .01; Figure 4D).

### 3.5 Plasma pNF‐H as a prognostic biomarker for somatomotor recovery

#### 3.5.1 Plasma pNF‐H and neuroscore

**FIGURE 5:**
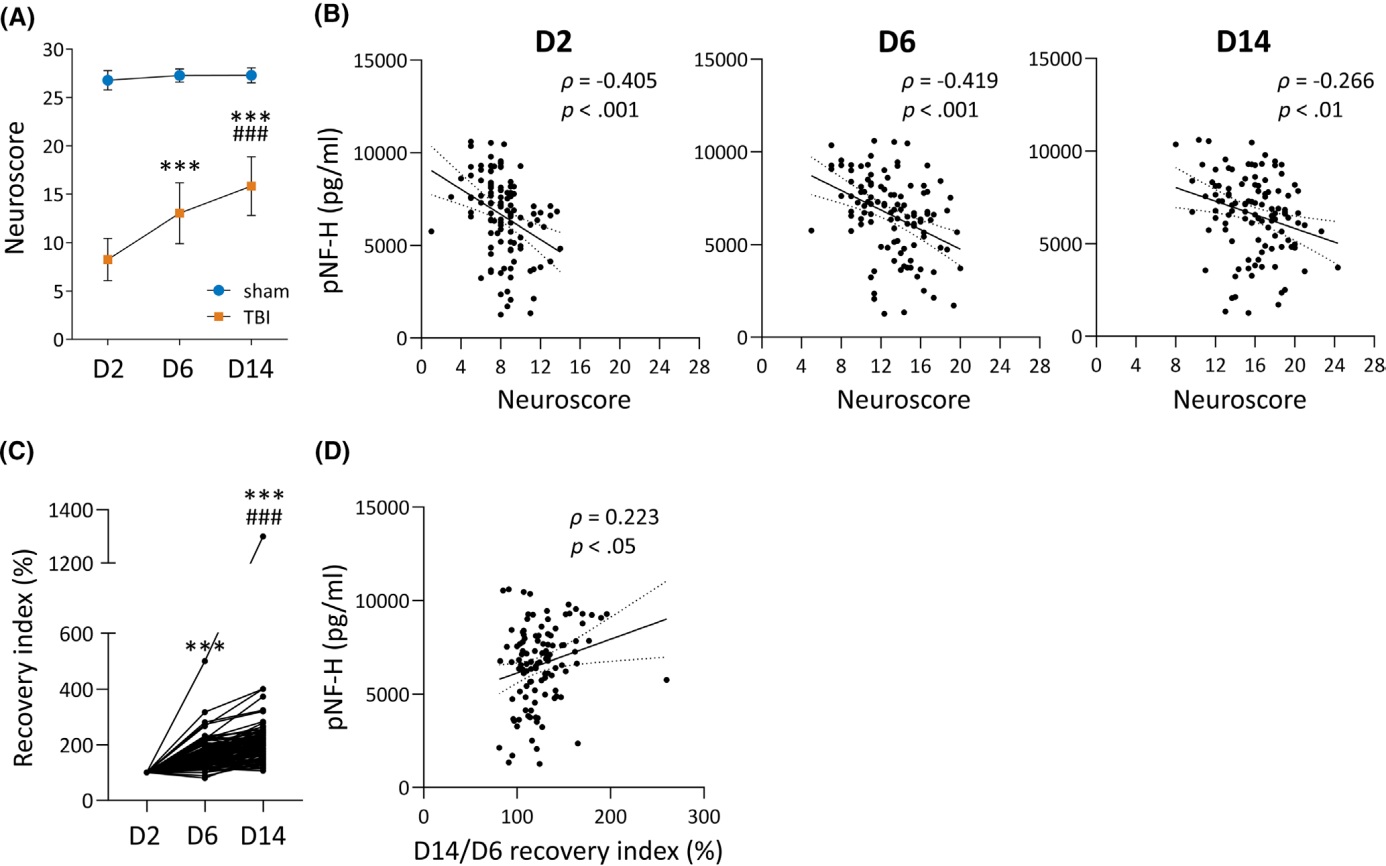
Plasma phosphorylated neurofilament heavy chain (pNF‐H) and somatomotor recovery. (A) Neuroscore recovery from day 2 (D2) to D14 after traumatic brain injury (TBI; *n* = 112) or sham operation (*n* = 21). Statistical significance: ***p < .001 compared to D2, ###*p* < .001 compared to D6 (Friedman test followed by Wilcoxon matched‐pairs signed rank test). (B) Spearman correlation (*ρ*) between plasma pNF‐H concentration on D2 and neuroscore on D2, D6, and D14 after TBI (*n* = 112). Dotted lines represent the 95% confidence interval. (C) Improvement of neuroscore (recovery index) for each TBI rat (*n* = 112) compared with the neuroscore on D2. ***p < .001 compared to D2, ###*p* < .001 compared to D6 (Friedman test followed by Wilcoxon matched‐pairs signed rank test). (D) Spearman correlation between plasma pNF‐H concentrations on D2 and late (D14/D6) recovery index (*n* = 112).

In the sham‐operated control rats (*n* = 21), the composite neuroscore was similar between testing days (Friedman test, *p* > .05). In the TBI group (*n* = 112), the composite neuroscore differed between testing days (Friedman test, *p* < .001; Figure 5A). On D2, the mean neuroscore was 8.3 ± 2.2 (range = 1.0–14.0, median = 8.0); on D6, 13.0 ± 3.1 (range = 5.0–20.0, median = 13.3, *p* < .001 compared with D2); and on D14, 15.8 ± 3.0 (range = 8.0–24.3, median = 16.0, *p* < .001 compared with D2 and D6). The higher the plasma pNF‐H concentration on D2 after TBI, the lower the neuroscore on D2 (*ρ* = −.405, *p* < .001), on D6 (*ρ* = −.419, *p* < .001), and on D14 (*ρ* = −.266, *p* < .01; Figure 5B).

#### 3.5.2 Plasma pNF‐H and recovery index

To assess whether plasma pNF‐H concentrations differed between rats with poor or good recovery, we calculated the recovery index (RI) for each TBI rat (Figure 5C). The average D14/D2 RI (overall recovery) of the 112 rats with TBI was 210% ± 117% (range = 107%–1300%). Of the 112 rats, 46% (52/112) had a good long‐term recovery (D14/D2 RI > 200%) and 54% (60/112) had a poor long‐term recovery (RI ≤ 200%). The average D6/D2 RI (early recovery) was 166% ± 52% (range = 80%–500%). Of the 112 rats with TBI, 59% (66/112) rats had a good early recovery (D6/D2 RI > 150%) and 41% (46/112) had a poor early recovery (RI ≤ 150%). The average D14/D6 RI (late recovery) was 125% ± 26% (range = 81%–260%). Of the 112 rats with TBI, 88% (98/112) had a good late recovery (D14/D6 RI >100%) and 12% (14/112) had a poor late recovery (RI ≤ 100%). Plasma pNF‐H concentrations on D2 did not differ between rats with (1) good or poor overall recovery, (2) good or poor early recovery, or (3) good or poor late recovery (all *p* > .05). Plasma pNF‐H concentrations on D2 did not correlate with D14/D2 or D6/D2 RIs. The higher the plasma pNF‐H concentration on D2, the higher the late (D14/D6) RI (*ρ* = .223, *p* < .05; Figure 5D). ROC curve analysis indicated that plasma pNF‐H concentration did not differentiate the good versus poor overall recovery, early recovery versus no early recovery, or late recovery versus no late recovery groups (*p* > .05).

### 3.6 Plasma pNF‐H as a prognostic biomarker for memory impairment

Our previous analysis indicated that a latency > 19.3 s to reach the platform on D3 of testing (D37 post‐TBI) in the Morris water maze differentiated cognitively impaired (CI+) from cognitively unimpaired (CI−) rats (AUC = .94).^47^, ^50^

#### 3.6.1 Plasma pNF‐H concentration and cognitive impairment

**FIGURE 6:**
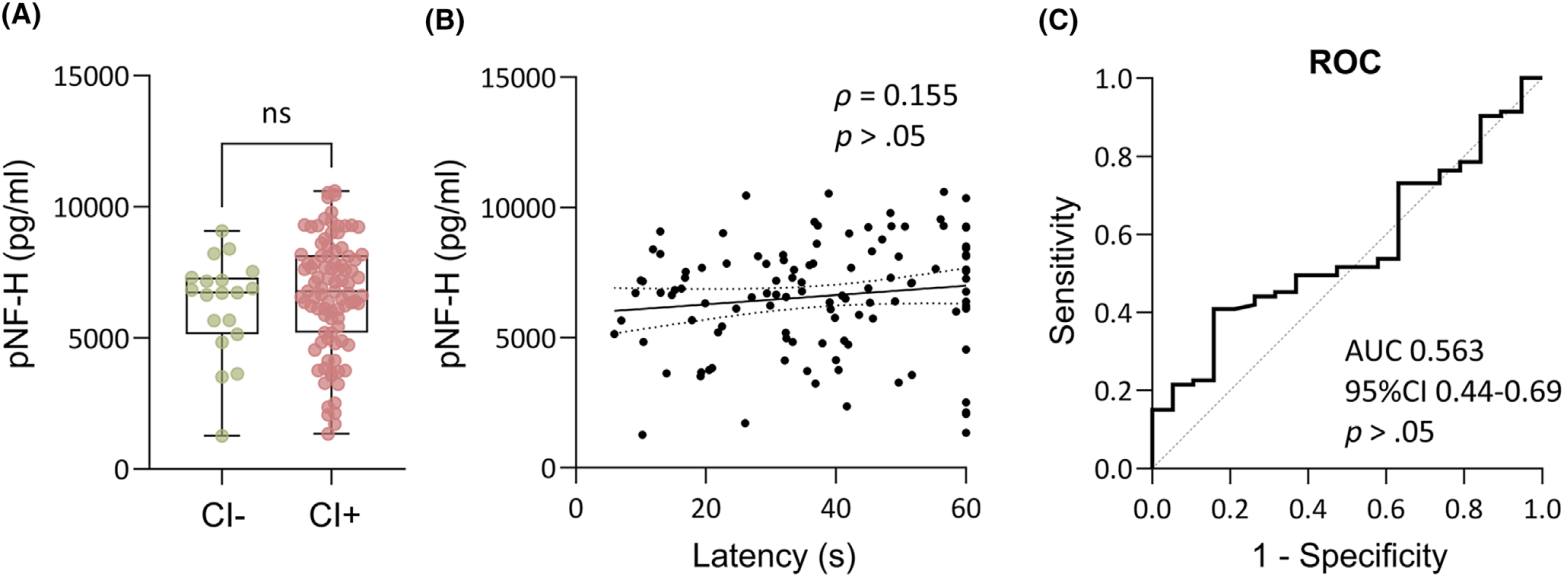
Plasma phosphorylated neurofilament heavy chain (pNF‐H) concentration and cognitive impairment. (A) Plasma pNF‐H concentrations were comparable between rats with (CI+; *n* = 93) or without (CI−; *n* = 19) cognitive impairment. (B) No correlation was detected between plasma pNF‐H concentration on day 2 (D2) and latency to find the hidden platform in the Morris water maze on D37 (Spearman correlation, *ρ*). Dotted lines represent the 95% confidence interval (95%CI). (C) Plasma pNF‐H concentration on D2 did not distinguish CI+ and CI− animals (area under the curve [AUC] = .56, *p* > .05). ns, not significant; ROC, receiver operating characteristic curve.

Plasma pNF‐H concentration on D2 after TBI did not differ between the CI+ (*n* = 92) and CI− groups (*n* = 19; Figure 6A). Plasma pNF‐H concentration on D2 did not correlate with the latency to find the hidden platform on D37 (*ρ* = .155, *p* > .05; Figure 6B). ROC curve analysis indicated that plasma pNF‐H concentrations did not distinguish the CI+ from CI− groups (AUC = .56, 95% CI = .44–.69, *p* > .05; Figure 6C).

## 4 DISCUSSION

This study is the first to assess whether plasma pNF‐H, a circulating marker of axonal injury in the brain, can be used as a prognostic biomarker for the development of epilepsy in an experimental model of human PTE. We had three major findings. First, high plasma pNF‐H concentration was associated with a large cortical lesion volume. Second, higher plasma pNF‐H concentration was associated with poor somatomotor performance but not with impaired hippocampus‐dependent spatial memory. Third, plasma pNF‐H concentration predicted the development of severe PTE.

### 4.1 Elevated plasma pNF‐H concentration reports on the severity of TBI‐induced cortical damage

An elevated circulating pNF‐H concentration after TBI is a marker of axonal injury in the brain and is proposed as one of the pathologies associated with PTE.^28^, ^51^, ^52^, ^53^, ^54^ A histologic study by Kanayama et al.^29^ showed pNF‐H expression in the perilesional cortex in perikarya, dendrites, and proximal axons as early as 6 h after severe lateral FPI in rats, increasing by 24 h. Our preliminary study indicated elevated plasma pNF‐H concentrations at 24 h after severe lateral FPI. The concentration increased severalfold by 48 h postinjury, remaining at the same level at 72 h, and decreasing thereafter by 7 days. Similarly, after controlled cortical impact (CCI) in rats, elevated serum pNF‐H concentrations were detected at 6 h postinjury, peaking at 48 h and reducing thereafter during the 168‐h follow‐up.^26^ In our EPITARGET cohort, plasma was sampled 48 h postinjury, representing peak plasma pNF‐H expression postinjury.

The average plasma pNF‐H concentration in naïve rats and in sham‐operated experimental controls collected at 48 h from tail vein blood was <LOD (23.5 pg/mL) of the ELISA kit in most cases. These data correlated with pNF‐H concentrations in serum prepared from the jugular vein blood of naïve and sham‐operated controls at 1 h, 4 h, and 24 h after lateral FPI.^35^ Interestingly, unlike NF‐L, pNF‐H concentration did not differ between naïve and craniectomized sham‐operated controls.^50^ This may relate to sensitivities of the two protein markers to detect mild lesions or of the analysis technologies (ELISA vs. single‐molecule array).

We found a 225‐fold increase in plasma pNF‐H concentration 48 h after severe lateral FPI compared with sham‐operated controls, reaching a median concentration of 6768 pg/mL. Yang et al.^35^ reported an increase in serum pNF‐H (to 300–1200 pg/mL at 24 h) after moderate lateral FPI as well as after CCI‐induced TBI (200–800 pg/mL) or penetrating ballistic brain injury (50–200 pg/mL). Karesioglu et al.^55^ reported a temporary 16% increase in serum pHF‐H concentrations 2 h postinjury, reaching 665 pg/mL and normalizing by 6 h, in the Marmarou weight‐drop model. Thus, an increase in circulating pNF‐H is rather consistent across TBI models known to lead to hyperexcitability or epilepsy.^56^ The magnitude of the change, however, is model‐ and impact severity‐dependent.

We also found a positive correlation between the plasma pNF‐H concentration and the severity of acute and chronic cortical damage: the greater the volume of T2 signal abnormality in MRI or cortical lesion area in histologic sections, the higher the plasma pNF‐H concentration. This is consistent with a previous experimental study suggesting that impact severity affects plasma NF‐H concentration.^26^ Consistently, clinical studies have demonstrated an association between circulating pNF‐H concentration and lesion severity. For example, Gatson et al.^27^ reported that in patients with mild TBI, the lower the Glasgow Coma Scale score, the greater the serum pNF‐H concentration. Furthermore, patients with computed tomography (CT)‐positive mild TBI had three‐ to fourfold higher serum pNF‐H concentrations than CT‐negative patients.^27^ An increased plasma pNF‐H concentration associates with microvascular injury after TBI,^57^ which is one of the pathologies associated with PTE.^58^

Taken together, both preclinical and clinical data suggest that circulating pNF‐H concentration correlates with the severity of TBI and can report on lesion severity and the presence of epileptogenic pathologies. Further studies are needed to assess the link between plasma pNF‐H levels and damage to specific white matter tracts during posttraumatic epileptogenesis.

### 4.2 Elevated plasma pNF‐H concentration as prognostic biomarker for the development of severe PTE

In TBI rats undergoing epileptogenesis, pNF‐H concentration on D2 was on average 15% higher than that in rats that did not develop epilepsy over the next 6 months (defined as the occurrence of one unprovoked late seizure^59^). As expected based on previous prognostic biomarker studies on TBI comorbidities,^60^ the epileptogenesis effect of plasma pNF‐H levels was substantially less than the injury effect, still separating the groups with an AUC of .647 and a cutoff value of 6773 pg/mL.

Sixty‐eight percent of the rats with epilepsy had ≥3 seizures per month and 36% had seizure clusters, indicating severe epilepsy in a subpopulation of rats with PTE. Consequently, the 30% elevation in the plasma pNF‐H concentrations in TBI+ rats with ≥3 seizures/month compared to TBI+ rats with <3 seizures/month separated the groups with an AUC of .784 with a cutoff value of 7672 pg/mL. Interestingly, we found a separation of animals with ≥3 seizures or a seizure cluster from the rest of the TBI animals (AUC = .732). Rats with milder epilepsy had pNF‐H levels comparable to that of the TBI− animals. This suggests that the brain injury produced was more severe in animals that developed severe epilepsy as compared to those with milder disease. This is in agreement with the epidemiological studies, showing that the risk of epilepsy increases with increasing severity of TBI.^5^, ^6^ As suggested by high NPVs, lower pNF‐H levels on D2 can rule out the cases that will not be at risk of developing PTE or severe PTE. This supports the idea that pNF‐H assay could be useful for stratification of high‐risk subjects for epileptogenesis studies by exclusion of cases at lower risk of PTE. This would already be a step forward for design of laborious powered preclinical and/or clinical treatment trials to combat posttraumatic epileptogenesis.

Our data demonstrate that plasma pNF‐H is a promising candidate for predicting the development of severe epilepsy after TBI. Further studies are needed, including more sampling time points to explore the value of pNF‐H as a prognostic biomarker for PTE in other TBI models and in human TBI cohorts.

### 4.3 Acute plasma pNF‐H concentration and epilepsy‐related comorbidities

Comorbidities, including those affecting physical and behavioral health and cognitive performance, are reported in up to 50% of patients with moderate to severe TBI.^61^ In a subpopulation of subjects, these impairments co‐occur with PTE.^62^

Our data show that high plasma pNF‐H concentration was associated with acute somatomotor impairment after lateral FPI but not with later somatomotor recovery. Moreover, acute plasma pNF‐H concentration did not differentiate rats that did or did not develop cognitive impairment. Studies on the value of pNF‐H as a prognostic biomarker for functional outcome in humans are limited. Some studies suggest that serum pNF‐H concentrations are greater in patients with a poor outcome compared to those with a good outcome,^63^, ^64^ but other studies report no associations between circulating pNF‐H concentration and medical or psychiatric comorbidities.^65^

Taken together, elevated plasma pNF‐H concentration shows some selectivity as a prognostic biomarker for post‐TBI morbidities, as it reports on the risk of developing PTE but not somatomotor recovery or cognitive decline.

## 5 CONCLUSIONS

This is the first report demonstrating the promise of circulating pNF‐H as a prognostic biomarker for severe PTE but not for milder PTE. Importantly, the pNF‐H concentration is normally very low, the concentration is easy to measure, the assay is affordable (~€20/sample), and marker concentrations peak at 2–3 days after injury, providing a feasible time window for management of blood sampling after injury. These data provide preclinical evidence for exploring the potential of pNF‐H as a prognostic biomarker for PTE in clinical studies.

## AUTHOR CONTRIBUTIONS

Asla Pitkänen and Noora Puhakka designed the study. Ivette Banuelos, Mette Heiskanen, Eppu Manninen, Pedro Andrade, and Noora Puhakka set up the methodologies. Ivette Banuelos, Mette Heiskanen, Eppu Manninen, Pedro Andrade, Noora Puhakka, and Elina Hämäläinen performed the experiments. Ivette Banuelos, Mette Heiskanen, Noora Puhakka, Eppu Manninen, and Elina Hämäläinen analyzed the data.. Pedro Andrade and Asla Pitkänen analyzed the EEG data. Eppu Manninen analyzed the MRI data. Asla Pitkänen and Mette Heiskanen compiled the data and wrote the manuscript.

## FUNDING INFORMATION

This study was supported by the Academy of Finland, the Sigrid Juselius Foundation, and the European Union's Seventh Framework Program (FP7/2007‐2013) under grant agreement No. 602102 (EPITARGET).

## CONFLICT OF INTEREST STATEMENT

The authors declare no conflict of interest. We confirm that we have read the Journal's position on issues involved in ethical publication and affirm that this report is consistent with those guidelines.

## Supporting information


Data S1.

## Data Availability

The data that support the findings of this study are available on request from the corresponding author. The data are not publicly available due to privacy or ethical restrictions.
