# Supplementary material for: Plasma neurofilament heavy chain is a prognostic biomarker for the development of severe epilepsy after experimental traumatic brain injury
Source: Epilepsia. 2024 Oct 14;65(12):3703–16. doi: 10.1111/epi.18149 (PMC11647440; doi:10.1111/epi.18149)
Supplement: Supplementary file 1 — Data S1. [file EPI-65-3703-s001.docx]

**Supporting document S1**

**1 | Materials and Methods**

**1.1 | Ethics**

All experiments were approved by the Animal Ethics Committee of the Provincial Government of Southern Finland and performed in accordance with the guidelines of the Directive 2010/63/EU of the European Parliament and of the Council of 22 September 2010 on the protection of animals used for scientific purposes.

**1.2 | Rats and induction of lateral fluid-percussion injury**

Adult male Sprague-Dawley rats were used (Envigo, The Netherlands). Average body weight at the time of injury or sham-operation was 356 g ± 14 g (median 356, range 326−419 g). Rats were housed in individual cages in a controlled environment (temperature 22 ± 1 °C, humidity 50%–60%, lights on 07:00–19:00), and had free access to food and water.

TBI was induced by lateral fluid-percussion injury (FPI) as previously described in detail^1^. Impact pressure in the EPITARGET cohort was adjusted to produce severe TBI with an expected post-impact mortality of 20%–30% within the first 48 h. The mean impact pressure in the pNF-H cohort was 3.26 ± 0.08 atm (n=112, median 3.26 atm, range 3.03 – 3.44 atm). Time in apnea and the occurrence and duration of impact-related seizure-like behaviors were monitored and documented. The sham-operated experimental controls underwent the same anesthesia and surgical procedures without the induction of lateral FPI. Naïve rats did not undergo any surgical procedures.

**1.3 | Plasma sampling and analysis of plasma pNF-H concentration**

***Blood sampling.*** Blood was sampled from the tail vein at 48 h (range -183 to 176 min; **Fig. S3**) after injury or sham operation. Plasma was prepared and stored as previously described in detail^2^. Briefly, rats were placed in an anesthesia chamber and anesthetized with 5% isoflurane. Anesthesia was maintained with 1%–2% isoflurane through a nose mask. Blood was drawn from the lateral tail vein into 2 Microtainer K_2_ EDTA-tubes (#365975, di-potassium ethylenediaminetetraacetic acid, BD Microtainer, BD Biosciences, Franklin Lakes, NJ, USA), 500 μl blood per tube, using a 24G butterfly needle. Within 1 h after blood sampling, blood tubes were centrifuged at 1300 x g for 10 min at 4°C (5417R Eppendorf Biotools). Plasma aliquots of 50 µl were carefully collected, pipetted into 0.5-ml Protein LoBind tubes (#022431064, Eppendorf AG, Hamburg, Germany), and stored at -70°C.

***Analysis of plasma NF-H concentration***. The ELISA kit provided by BioVendor (Cat. No. RD191138300R; Brno, Czech Republic) was used to analyze the concentration of phosphorylated neurofilament heavy chain (pNF-H). Briefly, samples were thawed, and diluted 1:6 with dilution buffer (DB) prior to the assay (40 µl of sample + 200 µl of DB), and they were kept in ice at a temperature ca. 4°C. Then, we performed the following procedure described in the kit’s manual with minor modifications. The concentration of pNF-H was calculated from the standard curve, and values were multiplied by the dilution factor (6x).

**1.4 | Behavioral tests**

**1.4.1 | Composite neuromotor score (neuroscore)**

The composite neuroscore test was used to measure the severity of somatomotor and vestibular deficits^3^. The tests were performed at D-6, D2, D6, and D14 (for details, see^1^. Briefly, the test included 7 parameters: (1) left and right forelimb flexion (2 parameters), (2) left and right hindlimb flexion (2 parameters), (3) left and right lateral pulsion resistance test (2 parameters), and (4) angle board standing test (1 parameter). The animals were scored from 0 (severely impaired) to 4 (normal) on an ordinal scale for each parameter, resulting in a composite neuroscore of 0–28.

To evaluate the rate of early (D2 to D6), late (D6 to D14), and overall (D2 to D14) somatomotor recovery, the recovery index was calculated for each TBI rat as a percentage difference in the neuroscore between the time points. Rats with a D6/D2 recovery index >150% were classified into the “good early recovery” group and those with an index ≤150% were classified into the “poor early recovery” group. Rats with a D14/D6 recovery index >100% were classified into the “good late recovery” group and those with an index of ≤100% were classified into the “poor late recovery” group. Rats with a D14/D2 recovery index >200% were classified into the “good overall recovery” group and those with an index ≤200% were classified into the “poor overall recovery” group.

**1.4.2 | Morris water-maze**

The Morris water-maze test was used to assess spatial learning and memory. The test, including a probe trial, was performed on D35–D39 after injury (for details, see^1^).

Cognitive performance of rats with TBI varied substantially on D35–D39, with some exhibiting severe impairment and others performing at the control level^1^. Therefore, we performed a cut-point analysis to categorize the TBI rats (n=118) into those that did or did not show memory impairment compared with sham-operated controls (n=23). To maximize the statistical power, the data used for the cut-point analysis were derived from the entire EPITARGET cohort. Based on the cut-off value of the latency (19.2 s) to find the hidden platform on day 3 of testing, we categorized each rat as “cognitively impaired” (CI+) or “cognitively unimpaired” (CI-).

**1.5 | Magnetic resonance imaging (MRI) for analysis of cortical lesion volume**

Details of the quantitative T_2_ MRI cortical lesion volume analysis were described previously^4^. The rats were imaged on D2, D7, and D21 after injury (**Fig. 1**). Briefly, multi-slice-multi-echo spin-echo images were acquired with a 7-Tesla Bruker PharmaScan MRI scanner (Bruker BioSpin MRI GmbH). The echo times were 14.6, 29.2, 43.8, 58.4, 73.0, and 87.6 ms and the repetition time was 3016 ms. Voxel size was 200 x 200 x 500 µm^3^. T_2_ relaxation time was estimated for each voxel using a monoexponential T_2_ signal model. Large cortical lesions made standard image registration-based approaches for outlining the cortical region of interest inaccurate. Therefore, the injured cortex was outlined manually for each rat and time point. Voxels within the range 45 ms ≤ T_2_ ≤ 55 ms were defined as having normal T_2_, with the lower limit equal to the 2.5^th^ percentile and the upper limit to the 97.5^th^ percentile of cortical voxels in the sham-operated control group. Values outside that range were classified as abnormal. For each animal, the total volume of abnormal voxels was computed to estimate the cortical lesion volume.

**1.6 | Video-EEG monitoring**

At 5 months post-TBI (D147), rats were anesthetized and implanted with 3 skull electrodes. Starting on D154 (1 week after electrode implantation), rats underwent continuous (24/7) video-EEG (vEEG) monitoring for 4 weeks to diagnose PTE (for details, see^1^). Rats were defined as having epilepsy if at least 1 unprovoked electrographic seizure was detected.

**1.7 | Histology and unfolded maps**

***Perfusion.*** To assess the location and extent of the FPI and exclude non-TBI related epileptogenic lesions such as abscesses, rats were intracardially perfused for histology after completing the vEEG (D182). Briefly, rats were deeply anesthetized with pentobarbital (60 mg/kg, i.p.) and perfused transcardially with 0.9% NaCl followed by 4% paraformaldehyde in 0.1 M sodium phosphate buffer (pH 7.4). The brain was removed from the skull, fixed in 4% paraformaldehyde for 4 h, cryoprotected in 20% glycerol in 0.02 M potassium phosphate-buffered saline (KPBS, pH 7.4) for 24 h, frozen in dry ice, and stored at -70°C for further processing.

Frozen coronal sections of the brain were cut (25-µm thick, 1-in-12 series) using a Leica sliding microtome. The first series of sections was stored in 10% formalin at room temperature and used for thionin staining. Other series of sections were collected into tissue collection solution (30% ethylene glycol, 25% glycerol in 0.05 M PB) and stored at -20°C until processed.

***Nissl staining.*** The first series of sections was stained with thionin, cleared in xylene, and cover-slipped using Depex® (BDH Chemical, Poole, UK) as a mounting medium.

***Preparation of cortical unfolded maps.*** To assess the cortical lesion area and the damage to different cytoarchitectonic cortical areas after TBI, thionin-stained sections were digitized (40x, Hamamatsu Photonics, NanoZoomer-XR, NDP.scan 3.2). Unfolded cortical maps were prepared using digitized histologic sections as described in detail by Ndode-Ekane et al.^5^ and by applying in-house software from https://unfoldedmap.org adapted to the rat brain^6^.

**1.8 | Statistical analysis**

Data were analyzed using GraphPad Prism 9 (GraphPad Software, Boston, Massachusetts, USA) and RStudio (v. 1.1.463) by R (v. 4.0.2). Comparisons of ≥ 3 groups were performed using the Kruskal-Wallis test followed by Dunn’s *post hoc* multiple comparisons test. The Mann-Whitney U test was used to compare 2 unpaired groups. Evolution of the T_2_ signal abnormality volume and the composite neuroscore over the testing period was assessed with the Friedman test followed by a *post hoc* Wilcoxon test. Correlations were assessed with the Spearman rank correlation test (ρ). Receiver operating characteristic (ROC) analyses were performed using the pROC package (v. 1.18.0) in R. Statistical significance of the area under the curve (AUC) was assessed by the Mann-Whitney U test. Optimal cut-point in ROC analysis was determined using the cutpointr package (v. 1.1.1) in RStudio by maximizing the sum of sensitivity and specificity. A p-value < 0.05 was considered statistically significant.

1. **| Results**

**2.1 | Preliminary analyses - Sample quality (hemolysis) and optimizing the timing of sampling**

***Hemolysis.*** Our preliminary analysis indicated that plasma pNF-H concentration did not correlate with hemolysis as determined by measuring the absorbance at 414 nm by NanoDrop (**Fig. S1**). Consequently, hemolysis was not measured for the entire cohort.

***Temporal expression of plasma pNF-H after TBI.*** We assessed the temporal expression of pNF-H during the acute post-injury phase (24 h, 48 h, 72 h, D5, D7 post-injury or sham-operation) and found a relatively stable peak at 48–72 h post-injury (**Fig. S2**).

***Accuracy of the timing of blood sampling.*** In the whole animal group, the average deviation from the 48-h time point was -3 ± 80 min (median -12 min, range -183 to +176 min). Data in different groups are summarized in **Fig. S3**.

**2.2 | Injury severity, acute post-impact seizure-like behavior, and apnea duration**

***Impact pressure.*** Mean impact pressure was 3.26 ± 0.08 atm in the TBI group (n=112), with no difference between the TBI+ and TBI- animals (3.25 ± 0.08 *vs.* 3.26 ± 0.08, p>0.05). No correlation was detected between the impact pressure and plasma pNF-H concentration on day (D) 2 (p>0.05).

***Duration of apnea.*** Mean post-impact apnea duration on D0 (injury day) was 34 ± 17 s in the TBI group (n=112). Apnea duration on D0 correlated with the plasma pNF-H concentration on D2 (r=0.353, p < 0.001).

Apnea duration did not differ between the TBI+ (n=28, 34 + 14 s) and TBI- (n=84, 33 + 17 s) groups (p>0.05, Mann-Whitney *U* test). Apnea duration did not differ between the TBI rats with (n=10, 29 + 14 s) and without (n=102, 34 + 17 s) seizure clusters (p>0.05, Mann-Whitney). Also, apnea duration did not differ between the TBI rats with (n=19, 34 + 16 s) and without (n=93, 33 + 17 s) high seizure number (p>0.05, Mann-Whitney).

***Acute seizure-like behavior.*** In total, 16% (18/112) of the rats with TBI exhibited acute post-impact seizure-like behavior. In rats with acute seizure-like behavior on D0 (n=18), the pNF-H concentration on D2 was 117% of that in rats without acute seizure-like behavior (n=94) (7 518 ± 1 534 pg/ml *vs.* 6 410 ± 2 192 pg/ml, p < 0.05).

There was no association between acute seizure-like behavior and late epilepsy. Also, occurrence of acute seizure-like behavior was not any more common in animals that developed seizure clusters or high seizure frequency (all p>0.05, Fisher’s exact test).


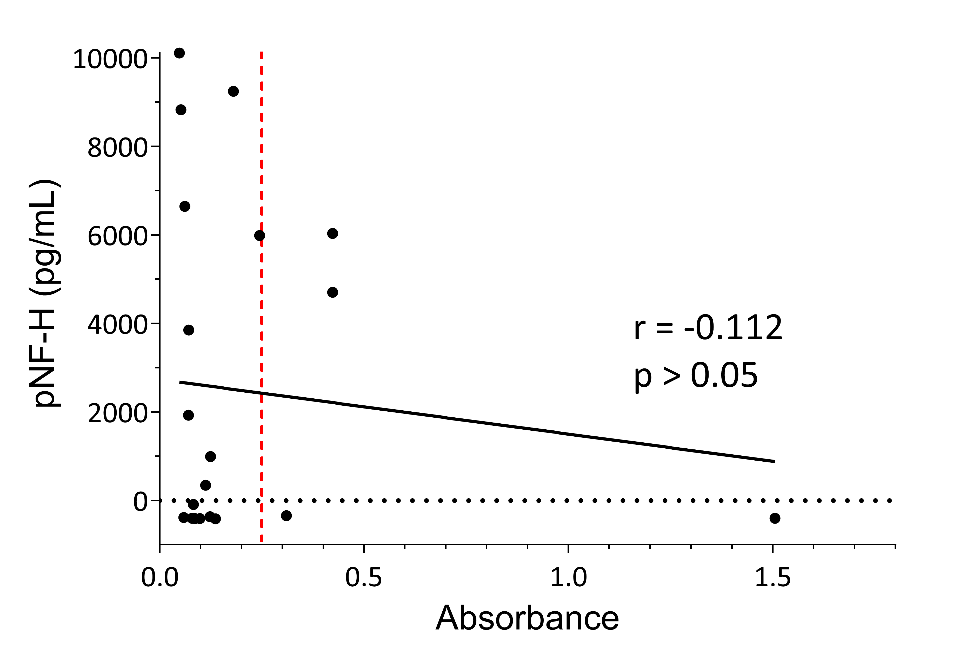


**Supporting Figure S1.** Spearman correlation analysis revealed no association between the plasma pNF-H concentration (y-axis) and absorbance at 414 nm (x-axis). Values >0.25 indicate hemolysis (red dashed line).


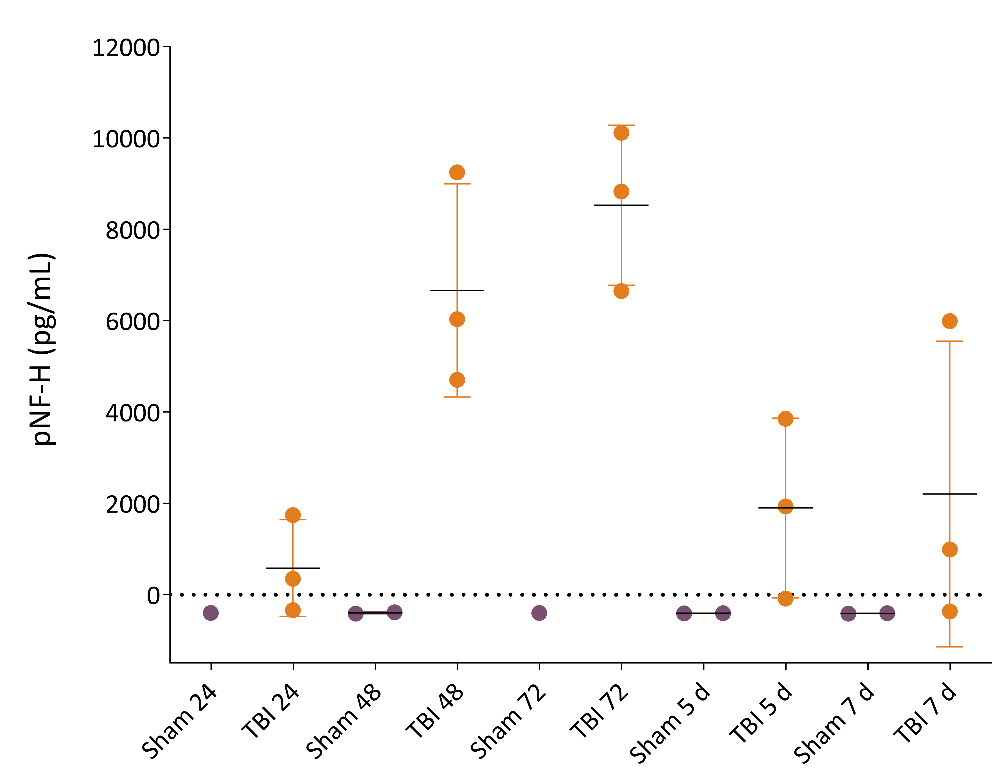


**Supporting Figure S2.** Preliminary analysis. Plasma pNF-H concentrations at different time points after lateral fluid-percussion-induced traumatic brain injury (TBI)(n=3, orange dots) or sham operation (n=2, purple dots). Note that a relatively stable peak of pNF-H concentrations occurred between 48 and 72 h after TBI, and the levels decreased thereafter.


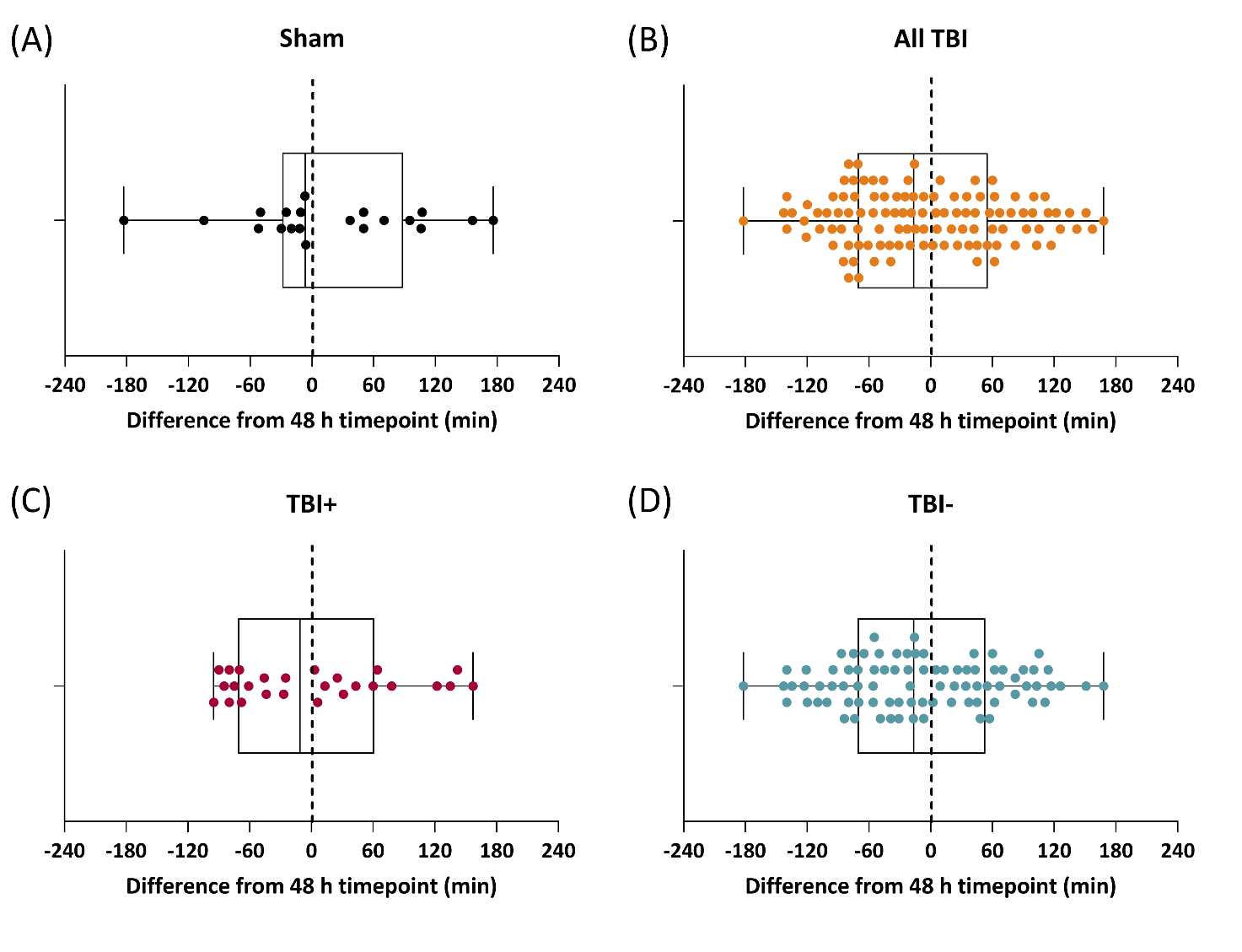


**Supporting Figure S3**. Box and whisker plots (whiskers: minimum and maximum, box: interquartile range, line: median) showing the timing of blood sampling relative to the anticipated 48-h timepoint (dashed line) on D2. Solid line indicates the median. Each dot represents one animal. **(A)** Sham-operated experimental controls (n=20, median -7 min, range -183 to +176 min). On average, blood samples were collected 17 ± 87 min after the 48 h time point. **(B)** TBI rats (n=110, average -7 ± 79 min, median -17 min, range -182 to +168 min) **(C)** Rats with epilepsy (TBI+)(n=26, average 1 ± 79 min, median -11 min, range -95 to +157 min). **(D)** Rats without epilepsy (TBI-)(n=84, average -9 ± 79 min, median -17 min, range -182 to +168 min).


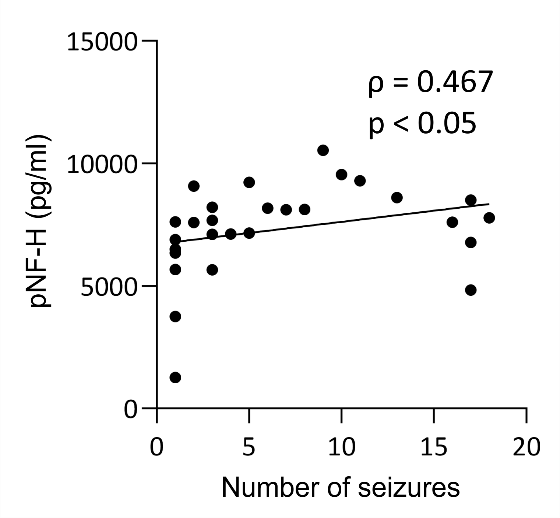


**Supporting Figure S4**. Spearman correlation analysis indicated that the higher the concentration of plasma pNF-H on D2 after TBI, the greater the seizure frequency during the 6^th^ post-TBI month (n=28, ρ = 0.467, p < 0.05).


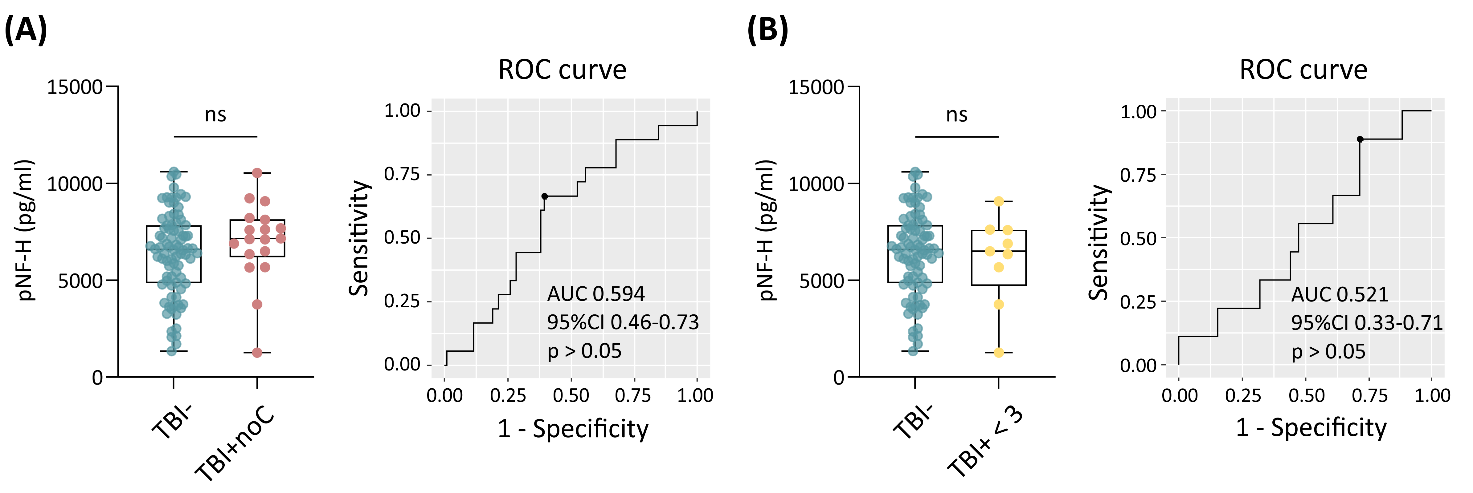


**Supporting Figure S5.** Plasma pNF-H levels in TBI rats without epilepsy (TBI-) and in TBI rats with mild epilepsy. **(A)** Plasma pNF-H concentration did not differ between TBI- rats (n=84) and TBI+ rats without seizure clusters (TBI+noC, n=18) (6 347 ± 2 168 pg/ml vs. 6 973 ± 2 077 pg/ml, p>0.05, Mann-Whitney test). Accordingly, ROC analysis showed that pNF-H concentration did not differentiate between TBI- and TBI+noC rats. **(B)** Plasma pNF-H levels did not differ between the TBI- rats (n=84) and the TBI+ rats that had < 3 seizures (n=9) (6 347 ± 2 168 pg/ml vs. 6079 ± 2325 pg/ml, p>0.05, Mann-Whitney test). ROC analysis showed that pNF-H concentration did not differentiate between TBI- rats and TBI+ rats with < 3 seizures. ***Abbreviations*:** AUC, area under the curve; CI, confidence interval; ROC, receiver operating characteristic; TBI-, TBI rats without epilepsy; TBI+, TBI rats with epilepsy; TBI+noC, TBI+ rats without seizure clusters.

| **Table S1.** Sensitivity, specificity, PPV, NPV and accuracy of D2 plasma pNF-H levels as a prognostic biomarker for different epilepsy outcomes. | | | | | | |
| --- | --- | --- | --- | --- | --- | --- |
| **Comparison** | **Sensitivity** | **Specificity** | **AUC** | **PPV** | **NPV** | **Accuracy** |
| TBI+ (28) vs. TBI- (84) | 75% | 58% | 0.65* | 38% | 88% | 63% |
| Clusters (10) vs. No-clusters (102) | 80% | 70% | 0.73* | 21% | 97% | 71% |
| HIGH TBI+ Sz number (19) vs. TBI+ LOW seizure number (9) | 63% | 89% | 0.78** | 92% | 53% | 71% |
| HIGH TBI+ Sz number (19) vs. all TBI LOW seizure number (93) | 89% | 58% | 0.73** | 30% | 96% | 63% |
| Number of animals in each group is in parenthesis. Statistical significances: *, p<0.05; **, p<0.01. ***Abbreviations:*** D, day; NVP, negative predictive value; PPV, positive predictive value; Sz seizure; TBI, traumatic brain injury; TBI rats without epilepsy; TBI+, TBI rats with epilepsy, TBI-, TBI rats without epilepsy. | | | | | | |

**REFERENCES**

1. Lapinlampi N, Andrade P, Paananen T, Hämäläinen E, Ekolle Ndode‐Ekane X, Puhakka N, et al. Postinjury weight rather than cognitive or behavioral impairment predicts development of posttraumatic epilepsy after lateral fluid‐percussion injury in rats. Epilepsia [Internet]. 2020; 61(9):2035–52. Available from: https://onlinelibrary.wiley.com/doi/10.1111/epi.16632

2. Heiskanen M, Jääskeläinen O, Manninen E, Das Gupta S, Andrade P, Ciszek R, et al. Plasma Neurofilament Light Chain (NF-L) Is a Prognostic Biomarker for Cortical Damage Evolution but Not for Cognitive Impairment or Epileptogenesis Following Experimental TBI. Int J Mol Sci [Internet]. 2022; 23(23). Available from: http://www.ncbi.nlm.nih.gov/pubmed/36499527

3. McIntosh TK, Vink R, Noble L, Yamakami I, Fernyak S, Soares H, et al. Traumatic brain injury in the rat: characterization of a lateral fluid-percussion model. Neuroscience [Internet]. 1989 [cited 2016]; 28(1):233–44. Available from: http://www.ncbi.nlm.nih.gov/pubmed/2761692

4. Manninen EM, Chary K, Lapinlampi N, Andrade P, Paananen T, Sierra Lopez A, et al. Early increase in cortical T2 relaxation is a prognostic biomarker for the evolution of severe cortical damage, but not for epileptogenesis, after experimental traumatic brain injury. J Neurotrauma [Internet]. 2020; :neu.2019.6796. Available from: https://www.liebertpub.com/doi/10.1089/neu.2019.6796

5. Ekolle Ndode-Ekane X, Kharatishvili I, Pitkänen A. Unfolded Maps for Quantitative Analysis of Cortical Lesion Location and Extent after Traumatic Brain Injury. J Neurotrauma [Internet]. 2017; 34(2):459–74. Available from: http://www.ncbi.nlm.nih.gov/pubmed/26997032

6. Ciszek R, Andrade P, Tapiala J, Pitkänen A, Ndode-Ekane XE. Web Application for Quantification of Traumatic Brain Injury-Induced Cortical Lesions in Adult Mice. Neuroinformatics. 2020; 18(2).
